# A Terrestrial Microbial Fuel Cell for Powering a Single-Hop Wireless Sensor Network

**DOI:** 10.3390/ijms17050762

**Published:** 2016-05-18

**Authors:** Daxing Zhang, Yingmin Zhu, Witold Pedrycz, Yongxian Guo

**Affiliations:** 1 School of Mechano-Electronic Engineering, Xidian University, Xi’an 710071, China; ymzhu@xidian.edu.cn (Y.Z.); wpedrycz@ualberta.ca (W.P.); 2 Department of Electrical and Computer Engineering, University of Alberta, Edmonton, AB T6G 2R3, Canada; 3 Department of Electrical and Computer Engineering, Faculty of Engineering, King Abdulaziz University, Jeddah 21589, Saudi Arabia; 4 Institute of Engineering Thermophysics, Chinese Academy of Sciences, Beijing 100190, China; guoyongxian@iet.cn

**Keywords:** terrestrial microbial fuel cell, wireless sensor network, energy harvesting, power management

## Abstract

Microbial fuel cells (MFCs) are envisioned as one of the most promising alternative renewable energy sources because they can generate electric current continuously while treating waste. Terrestrial Microbial Fuel Cells (TMFCs) can be inoculated and work on the use of soil, which further extends the application areas of MFCs. Energy supply, as a primary influential factor determining the lifetime of Wireless Sensor Network (WSN) nodes, remains an open challenge in sensor networks. In theory, sensor nodes powered by MFCs have an eternal life. However, low power density and high internal resistance of MFCs are two pronounced problems in their operation. A single-hop WSN powered by a TMFC experimental setup was designed and experimented with. Power generation performance of the proposed TMFC, the relationships between the performance of the power generation and the environment temperature, the water content of the soil by weight were measured by experiments. Results show that the TMFC can achieve good power generation performance under special environmental conditions. Furthermore, the experiments with sensor data acquisition and wireless transmission of the TMFC powering WSN were carried out. We demonstrate that the obtained experimental results validate the feasibility of TMFCs powering WSNs.

## 1. Introduction

Microbial fuel cells (MFCs), converting chemical energy from organic compounds to electrical energy through catalytic reactions of microorganisms, can be envisioned as archetypical microbial Bioelectrochemical Systems (BESs). MFCs have attracted a large amount of interest in the past decade because they can generate electric power while treating waste. Unlike other fuel cells, MFCs can continuously generate power at normal temperature, atmospheric pressure, and neutral pH value without any additional maintenance. In addition, the only byproducts are CO_2_ and H_2_O, which do not require additional handling. Thus, MFCs have been widely considered as one of the devices harvesting sustainable energy [1,2,3,4,5]. In America, it causes about 1.5% of the municipal energy consumption for waste water treatment. In some industrialized countries, the wastewater treatment results in about 10% of the municipal energy consumption [1]. At the same time, the energy content of municipal wastewater is considered to be nine times higher than the energy demand necessary for its treatment [6]. There is an enormous potentiality and great prospects for development to generate electric power through treating wastewater.

Research exploiting the bioelectrocatalytic activity of living microorganisms to generate electric power have a history of 100 years [7]; however, MFCs as a novel technology is still in development. Systematic MFC development started only a decade ago [8]. At present, the aquatic microbial fuel cells (AMFCs) power generation properties have been verified, and many efforts were made to improve the power generation properties and the energy harvesting efficiency [1,4,9,10,11,12,13,14,15,16,17,18,19,20]. AMFCs are validated for powering small electronic devices [21,22,23,24,25,26,27,28,29,30,31,32,33]. However, AMFCs must be inoculated and work in the water environment [1,2,3,4,5], which limits the MFCs’ application fields. Terrestrial microbial fuel cells (TMFCs) can be inoculated and worked on land [34,35,36], which can overcome the disadvantages of AMFCs and extend the MFCs’ application range.

The concept of TMFC was first presented in 2010. A TMFC setup was designed to power a wireless environmental sensor network. Unfortunately, it was found that more than two-thirds of sensor nodes worked improperly. More importantly, no certain conclusion about the nature of this problem was formulated [34]. In successive years, little progress has been made in the study of TMFCs [35,36]. TMFCs cannot be widely adopted because of several pronounced challenges. Among them, the low power density and low reliability are two open problems [34,35,36]. As a result, a Power Management System (PMS) is usually required to connect a TMFC to the load. Low efficiency is one of the main problems in the PMS development. Much effort has been made to develop an appropriate PMS [11,16,19,25,28,29,33]. So far, no universal PMS has been proposed. Some environmental factors affect the power density of TMFC, among them including temperature, soil bacteria, and biological content [34,35,36]. However, at present, the relationships between the power generation performance and environmental factors are still under discussion. Further research should be completed to arrive at sound conclusions.

Wireless sensor networks (WSNs), as the first of ten new technologies deeply affecting human future life, exhibit a tremendous value for a broad range of applications. They play an increasingly important role in military, industrial, and consumer applications [23,37,38,39,40]. However, the long-lasting power supply for WSN nodes remains one of the bottlenecks hampering their further rapid development. Power sources are one of the most important and influential factors determining the lifetime of WSN nodes, especially for remote environment monitoring applications. Reducing power consumption, improving the efficiency of power consumption, and developing new power supply methods are effective ways to solve this problem. WSN nodes powered by energy harvesting sources could theoretically have an eternal lifespan [38,39,40].

The findings in this regard are the ultimate subject of this study. In this study, our aim is to improve the performance of the proposed TMFC and develop a TMFC powering of a single-hop WSN node experimental setup. The intent is to come up with a new reliable method for powering the WSN node for a long time.

## 2. Results

### 2.1. Terrestrial Microbial Fuel Cell (TMFC) Performance

The performances of the proposed TMFC including the voltage between electrodes during inoculation, the open circuit voltage (OCV), and the polarization curve were reported through a series of experiments.

#### 2.1.1. Voltage between Electrodes in the Inoculation Experiment

**Figure 1:**
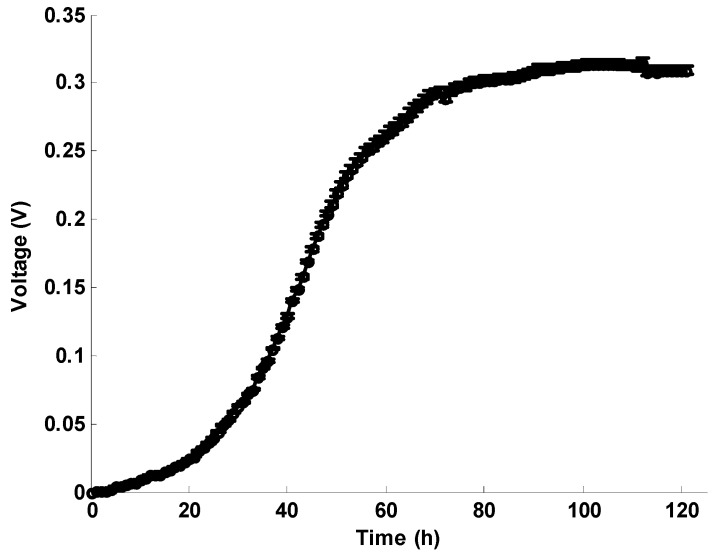
Voltage between electrodes in the inoculation experiment.

The voltage between electrodes in the inoculation experiment is shown in Figure 1. After injecting the medium, the voltage between the electrodes starts increasing. About 30 h later, a rapid voltage (current) increase is found. This phenomenon is also observed in our AMFC experiments, and it has also been verified in other papers [1,4,25]. About 80 h later, the voltage stabilizes at a value of about 0.3 V.

#### 2.1.2. Open Circuit Voltage

**Figure 2:**
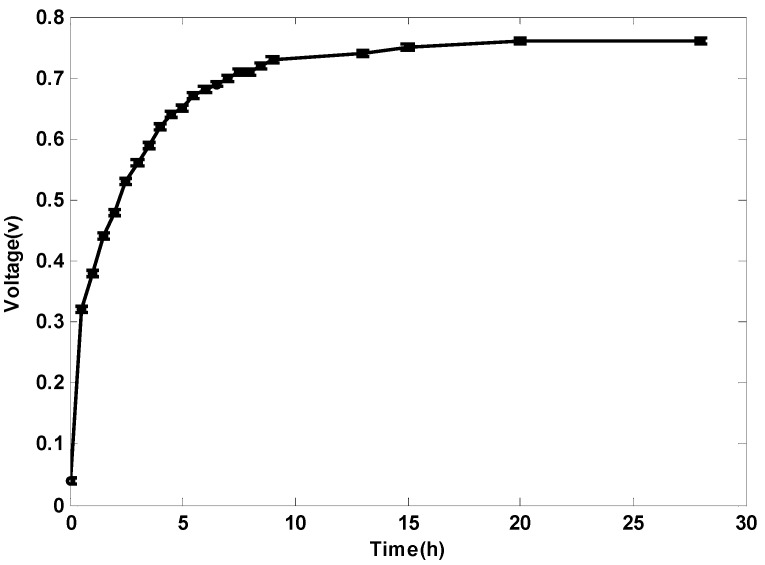
Open circuit voltage (OCV) of the proposed terrestrial microbial fuel cell (TMFC).

After the inoculation experiment, the resistor between electrodes was disconnected, and the OCV was tested; refer to Figure 2. It took about 25 h for the voltage between electrodes to become stable. The experiment can be implemented any time after inoculation. The OCV of the proposed TMFC is about 0.75 V, which is a reasonable value for MFCs [1].

#### 2.1.3. Polarization and Power Curves

**Figure 3:**
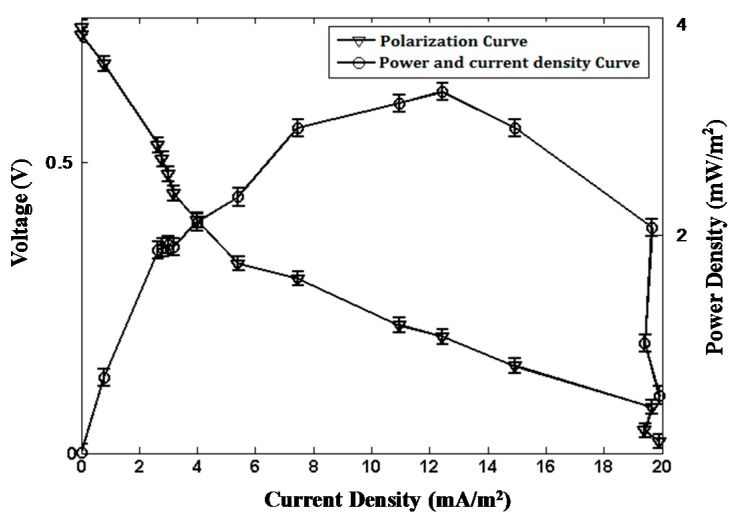
Polarization and power curves of the TMFC.

Polarization and power curves are the representative characteristics for the power generation performance of MFCs. These curves of the proposed TMFC (Figure 3) were obtained according to the procedure detailed in Materials and Methods.

The proposed TMFC has an OCV of about 0.75 V, and the maximum current density is about 20 mA/m^2^, which are reasonable values for typical MFCs [1]. The TMFC exhibited a maximum power density of roughly 3 mW/m^2^ of anode surface area, which is lower than the maximum power density of the AMFC established in our previous study [19]. No membrane is used in this study, which may lead to a low efficiency of the setup. Additionally, the activity of microorganisms in water is usually higher than in soil. These may be the reasons for the lower power density of the proposed TMFC. The lower power density leads to a longer duty cycle of the PMS and the WSN platform, which is not the key problem for a long-term monitoring application of WSNs. The advantages of our TMFC are the simple architecture and economical materials.

### 2.2. Relationship between the Proposed TMFC Properties and Environmental Factors

#### 2.2.1. Soil Moisture Content

**Figure 4:**
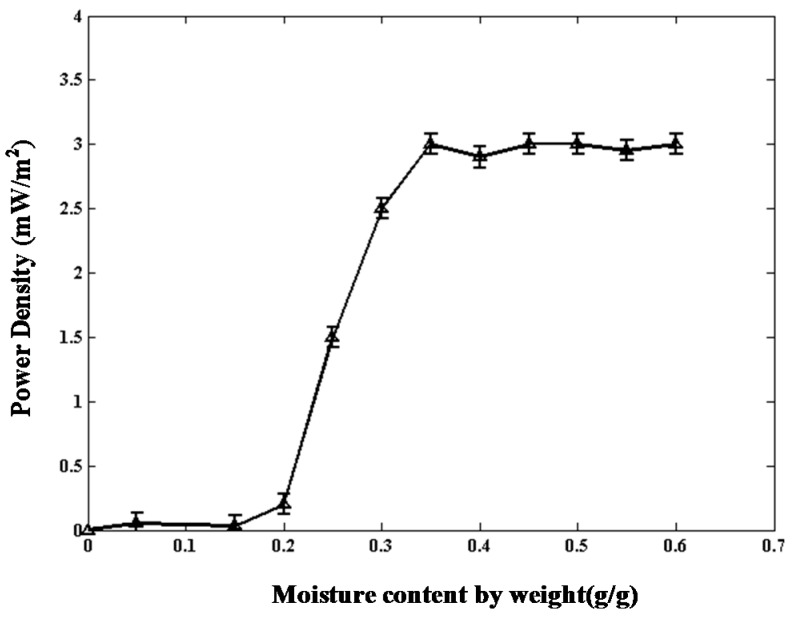
Relationship between the soil moisture content and the power density.

The relationship between the soil moisture content by weight and the output power density of the TMFC is recorded; refer to Figure 4. The output power density nears zero when the soil moisture content is below approximately 0.2 g/g. The power density increases with the increase of soil moisture content, while the soil water content is between 0.2 and 0.4 g/g. The output power density achieves the maximum when the soil moisture content approaches 0.4 g/g. The power density remains unchanged if the soil moisture content exceeds 0.4 g/g.

#### 2.2.2. Temperature

**Figure 5:**
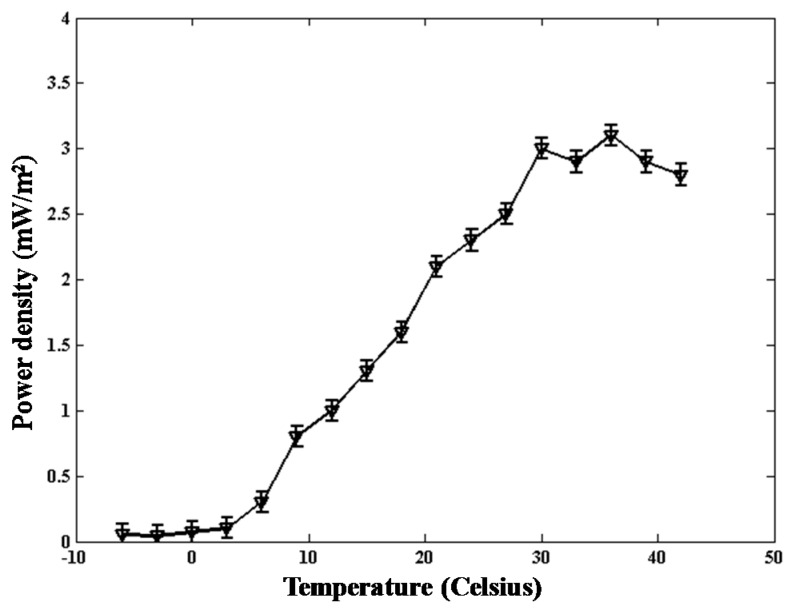
Relationship between the power density and temperature.

We recorded the relationship between temperature and the power density of the proposed TMFC. The result is shown in Figure 5. As shown in the figure, the output power density increases with the increase of temperature, while the temperature is below 36 °C. The output density begins to drop when the temperature is greater than 36 °C.

It is not convenient to maintain a specific temperature while running experiments. Noticeably, the output power density exhibits little difference between room temperature (about 25 °C) and 36 °C. Subsequently, other experiments were realized at room temperature, and the soil water content by weight is kept at about 0.4 g/g.

### 2.3. Voltages on Super-Capacitors in the Power Management System (PMS) Board

**Figure 6:**
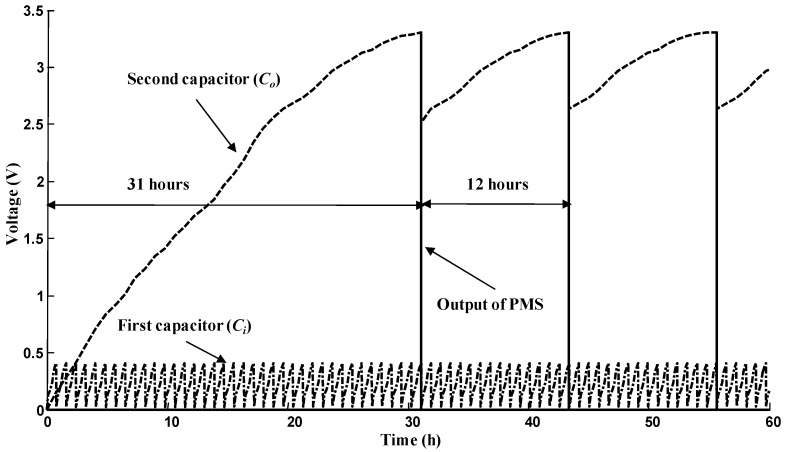
Voltage recorded on super-capacitors of the Power Management System (PMS) board.

In order to characterize the performance of the PMS, we recorded the voltages on the super-capacitors in the PMS board (Figure 6).

The PMS board works as in the literature [19]. The charge–discharge cycle of the first super-capacitor is about 0.85 h (±0.05). The stable duty cycle is about 12 h (±0.5).

### 2.4. Wireless Sensor Network (WSN) Application

**Figure 7:**
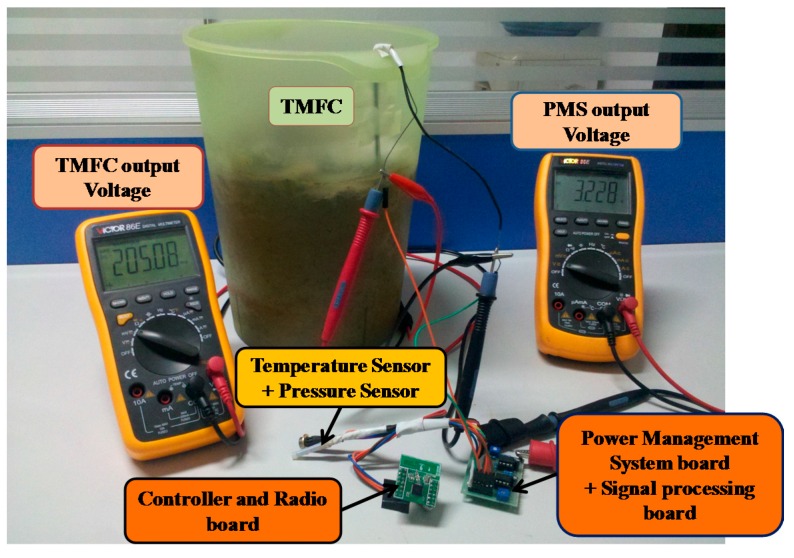
Photo of the experimental setup of the sensor node.

The sensor node experimental devices are shown in Figure 7. The signal processing board and controller and radio board are powered by the TMFC through the PMS board. The voltage of TMFC output (the voltage on the first super-capacitor) and the voltage of PMS output (the voltage on the second super-capacitor) are monitored by multimeters.

**Figure 8:**
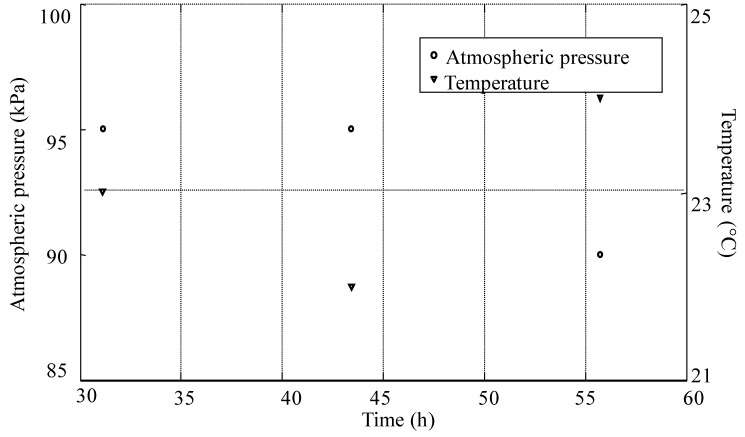
Sensor data received by access point.

The maximum output voltage of the PMS board is 3.3 V, which meets the working voltage requirement of the WSN board. However, the WSN board can transmit data only when the second super-capacitor (Co) stores enough energy, which depends on the value of the second super-capacitor (Co). As mentioned above, its capacitance is 100 mF (the choice of this value is based on experimental results [19]). The sensor node, powered by the TMFC, acquires and transmits data to the access point periodically. The sensor data that are received by the access point are transmitted to the PC via the RS232 interface. The sensor data saved on the PC are shown in Figure 8.

The access point receives three packets each period, which includes the temperature and atmospheric pressure information. The stable duty cycle is about 12 h, which is in accordance with the duty cycle of the PMS board. Because the experiments were implemented in the laboratory environment, the sensor data do not vary too much over time.

## 3. Discussion

Experimental results show that the WSN board works properly by being powered by the proposed TMFC reactor. Due to the low output voltage and low output power coming from the TMFC, the output voltage must be boosted, and the energy must be accumulated through the PMS board before powering the WSN board. Therefore, the WSN board can only work periodically. The duty cycle depends on the performance of the TMFC, the value of the super-capacitors on the PMS board, and the power consumption of the load. The environment temperature and the soil moisture content by weight are critical to the improvement of the performance of the TMFC.

It would be interesting to use TMFCs to power electronic devices for longer times and with shorter duty cycles. Improving the power generation properties of the TMFCs and/or developing a more efficient PMS could be effective methods. Future studies may include as follows: the determination of other influence factors, such as soil bacteria and biological content for TMFC power generation properties; and the development of WSN protocols while using the proposed TMFC.

## 4. Materials and Methods

### 4.1. Terrestrial Microbial Fuel Cell Device

**Figure 9:**
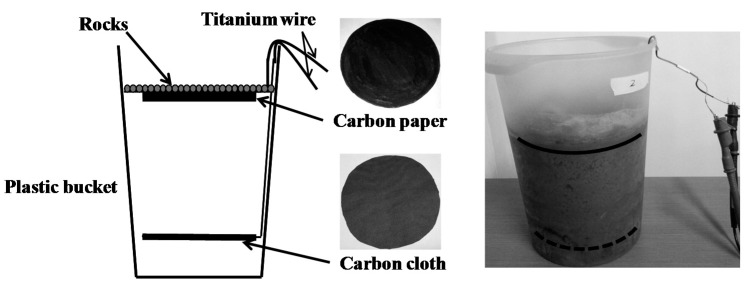
Proposed TMFC architecture.

A TMFC experimental device has been designed and constructed; see Figure 9. The TMFC reactor is a plastic bucket. The diameter is 150 mm at the bottom, and 190 mm at the top. It has a height of 260 mm. The anode material is carbon cloth (HCP330, Hesen, Shanghai, China), which is a disk of 160 mm diameter, and it is placed at a height of 45 mm from the bottom of the bucket. The cathode material is carbon paper (HCP030, Hesen, Shanghai, China), which is a disk that is 160 mm in diameter. The anode is covered with Pt-catalyst (XC-72, Fuel Cell Store, Boulder, CO, USA). The electrodes have a distance of 110 mm. On the top of the cathode, a layer of rocks is placed. This provides the deformation of the carbon paper. The electronic loads were connected to the anode and cathode by two titanium wires. We collected soil from the forest (Qinling Mountains, Xi’an, China) off the beaten path, which tends to be rich of microbes and nutriments.

### 4.2. Performance Evaluation of the TMFC

#### 4.2.1. Electrochemical Tests

The TMFC reactor is inoculated and works at room temperature (about 25 °C). In the inoculation experiments, an external 1-kΩ resistor is connected to the anode and cathode. In the beginning, the original soil (with a moisture content of about 0.15 g/g by weight) is used in the experiments. The output voltage of TMFC remained zero for more than seven days. Then, 1 L of DI water-based medium (with 500 mg glucose and 500 mg NaCl) was added to the TMFC reactor. Glucose is helpful in enhancing the nutrients of the soil, and NaCl is helpful in increasing the level of ions in the setup. Glucose and NaCl are not indispensable here. They are used to reduce the inoculation time. A rapid voltage (current) increase is observed, and the voltage then remains stable a few days later. This suggests that the inoculation process has been realized [1,11,19].

The voltage between anode and cathode was recorded using a digital multimeter (VC86E, VICTOR, Shenzhen, China). When the inoculation experiments were completed, the external 1-kΩ resistor was disconnected, and the OCV was measured using the digital multimeter. After the OCV reached the stable values, the polarization curve was produced as follows: A series of resistors were connected to the TMFC output with the resistance progressively changed from 10 kΩ to 50 Ω every 10 min. Ten minutes were sufficient for the TMFC reactor to reach the steady state after each resistance change [35]. The values of the output voltage and resistance of the resistors were recorded every time. The associated current was also calculated (by dividing the recorded voltage by the external resistance).

#### 4.2.2. Environmental Factors Effects on the Performance of TMFC

In order to improve the power generation performance of the proposed TMFC, the impact of the environmental factors (temperature and soil moisture content by weight) was analyzed. The environmental temperature was about 25 °C. The soil moisture content was recorded using a soil moisture tester (TZS-I, Top Instrument, Hangzhou, China). At first, soil with low water content was selected. The water content was increased by adding DI water into the reactor; this is more convenient than using an oven drying method. We waited about 1 h every time for the TMFC to reach a stable state. It is validated that this method has a lesser effect on the microorganism in the soil than oven drying technique.

The reactor was placed in a temperature humidity chamber (THS-B4H-100, KSON, New Taipei, Taiwan) to test the relationship between temperature and power density. The temperature was progressively changed from −6 to 42 °C with an interval of 3 °C. The soil moisture content was kept at about 0.4 g/g during the experiments. It indicated that maintaining at least one hour at every temperature is enough to produce the polarization curves.

### 4.3. WSN Platform Implementation

**Figure 10:**
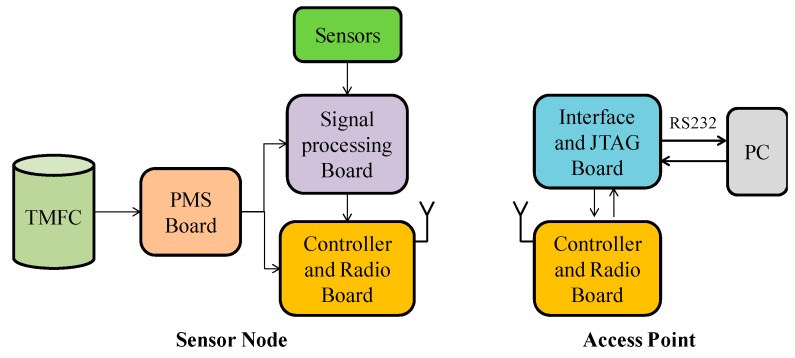
Overall block diagram of the Wireless Sensor Network (WSN) platform. JTAG, Joint Test Action Group.

The WSN platform comprises two parts: a sensor node and an access point. The sensor node consists of two sensors, a signal processing board, a controller and radio board, a TMFC reactor, and a PMS board. The access point includes a controller and a radio board (the same as used in the sensor node), a PC, an interface, and a Joint Test Action Group (JTAG) board. The overall block diagram of the WSN platform is shown in Figure 10.

#### 4.3.1. Sensors and Signal Processing Board

**Figure 11:**
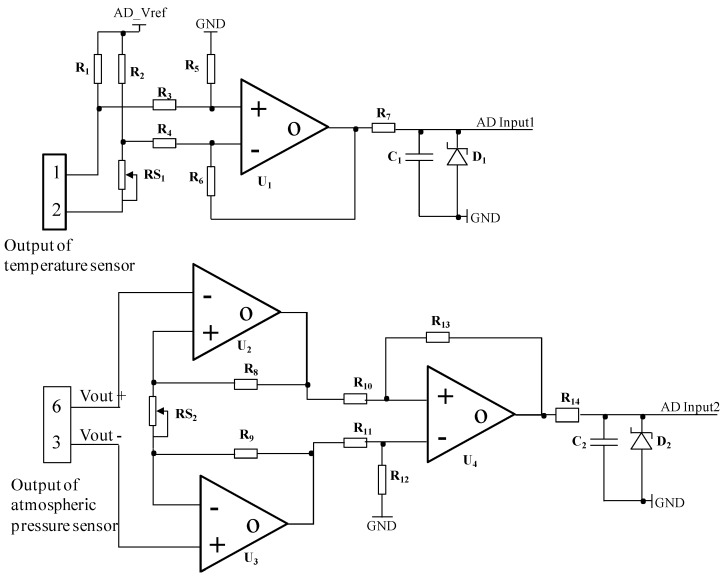
Schematic diagram of the signal processing board. GND, Ground.

Two sensors are selected for environmental monitoring. One is for temperature monitoring (WZP-010, Heraeus, Hanau, Germany), and the other is for atmospheric pressure monitoring (MPS30H0500AT, MEMStek, Wuxi, China). Because the output signals coming from the sensors are very weak and are easily affected by noise, a sensor signal processing board is designed to amplify the output signals of sensors. After amplifications, the signals are acquired by the controller. The main component of the signal processing board is a precision amplifier (OPA2340, TI, Dallas, TX, USA). It can operate at a voltage as low as 2.5 V. The schematic diagram of the signal processing board is shown in Figure 11.

The Net label AD_Vref is connected to the AD convertor’s reference voltage pin of the controller chip. AD Input1 and AD Input2 are connected to the AD convertor’s input0 pin and input1 pin of the controller chip, respectively. The adjustable resistor RS_1_ is used to adjust the output voltage of U_1_. The adjustable resistor RS_2_ is used to adjust the output voltage of U_2_ and U_3_, and then can adjust the output voltage of U_4_. The Zener diodes D_1_ and D_2_ are used to protect the AD convertor of the controller chip from overvoltage. The values of the parameters of the components shown in the diagram are reported in Table 1.

#### 4.3.2. Controller and Radio Board

**Figure 12:**
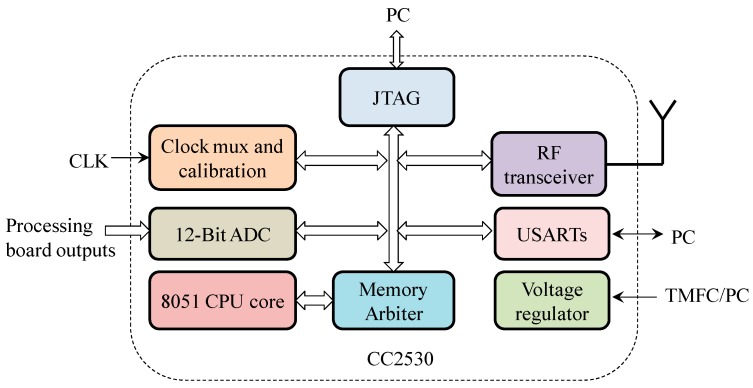
Overall diagram of the controller and the radio board. RF, Radio Frequency. CLK, Clock.

The controller and radio board were designed for data conversion, processing, and wireless transmission. A true system-on-chip solution for 2.4-GHz IEEE 802.15.4 and ZigBee application chip (CC2530F256, TI, Dallas, TX, USA) has been adopted. It includes a 2.4 GHz IEEE 802.15.4 compliant Radio Frequency (RF) transceiver, a high-performance and low-power 8051 microcontroller with code prefetch, and a 12-Bit ADC with eight channels. Thus, sensor data acquisition, processing, and wireless transmission can be realized on a single chip, which simplifies the circuit and reduces power consumption. The power consumption is about 90 mW, while sending data under 1 dBm. It also has two powerful USARTs with support for several serial protocols, which is convenient for the board to communicate with a PC. The overall diagram of the board is shown in Figure 12.

The controller and radio boards in sensor node and access point have the same structure. However, the controller and radio board at the sensor node is powered by the TMFC reactor through the PMS board, and the controller and radio board in the access point is powered by the PC via USB.

#### 4.3.3. PMS Board

**Figure 13:**
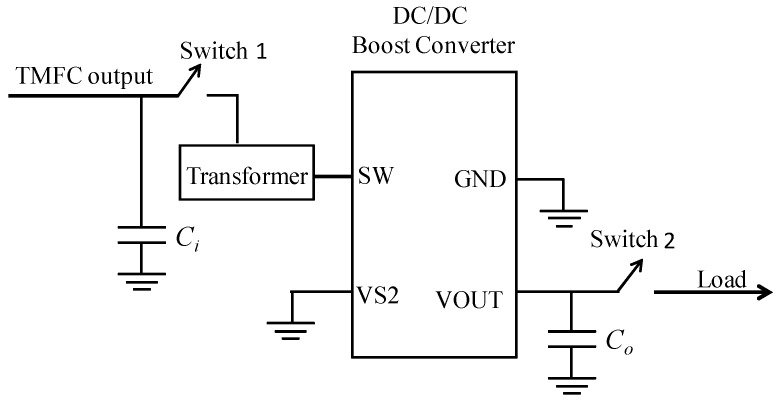
Principle diagram of the PMS board.

Because of the low output voltage and low output power density of TMFCs, it requires a PMS to connect a TMFC to the electronic load. A capacitor-transformer converter PMS board is designed based on our previous study [19]. The principle diagram of the PMS board is shown in Figure 13.

Two super-capacitors are used in the PMS circuit: One is directly connected to the TMFC output electrodes, and the other is connected to the DC/DC converter output pins. The first super-capacitor (*C_i_*) is used for accumulating power from the TMFC and driving the following DC/DC converter. This super-capacitor needs to be optimized. The optimal value of the first super-capacitor is 1F based on the method of our previous study [19,24]. The second super-capacitor (*C*_0_) is used for storing energy from the DC/DC converter and drives the electronic load. Its capacitance is determined based on the power consumption of the load. The value is 100 mF based on the experimental results of transmitting three packets in one duty cycle. In this application, LT3108 (Linear Technology, Milpitas, CA, USA) is selected as the DC/DC converter. When the voltage of the first super-capacitor *C_i_* reaches the turn-on voltage of Switch 1, Switch 1 closes. The first super-capacitor powers the rest of the PMS and the load as a power source. Similarly, when the voltage of the second super-capacitor *C*_0_ achieves the turn-on voltage of Switch 2, Switch 2 closes and the energy storing in the super-capacitor *C*_0_ starts to drive the load. The minimum acceptable input voltage of the board is 0.18 V, and the maximum output voltage is 3.3 V.

## Figures and Tables

**Table 1:** Values of the component parameters shown in Figure 11.

Name	Symbol	Value/Name of the Component
Variable Resistor	RS_1_, RS_2_	500 Ω
	R_1_, R_2_	500 Ω
	R_3_, R_4_	25 KΩ
Resistor	R_5_, R_6,_ R_8_, R_9_	100 KΩ
	R_7_, R_14_	1 KΩ
	R_10_, R_11,_ R_12_, R_13_	10 KΩ
Capacitor	C_1_, C_2_	10 nF
Zener Diode	D_1_, D_2_	IN4733
Amplifier	U_1_, U_2,_ U_3_, U_4_	OPA2340

## Abbreviations

MFCs	Microbial Fuel Cells
TMFCs	Terrestrial Microbial Fuel Cells
AMFCs	Aquatic Microbial Fuel Cells
WSNs	Wireless Sensor Networks
BESs	Bioelectrochemical Systems
OCV	Open Circuit Voltage
PMS	Power Management System
PC	Personal Computer
USB	Universal Serial Bus
JTAG	Joint Test Action Group
USART	Universal Synchronous Asynchronous Receiver Transmitter
RF	Radio Frequency
ADC	Analog to Digital Converter
DC/DC	Direct Current to Direct Current
